# Draft genome sequence of *Bacillus velezensis* CMAA1918 isolated from the rhizosphere of drought-tolerant plant *Neoglaziovia variegata*

**DOI:** 10.1128/mra.01378-25

**Published:** 2026-04-23

**Authors:** Gileno Vieira Lacerda-Júnior, Itamar Soares Melo

**Affiliations:** 1 Laboratory of Environmental Microbiology, Brazilian Agricultural Research Corporation - EMBRAPA 67838, Jaguariúna, São Paulo, Brazil; University of Wisconsin-Madison, Madison, Wisconsin, USA

**Keywords:** osmotolerant bacteria, *Neoglaziovia variegata*, *Bacillus velezensis*, drought stress, genomic features

## Abstract

We report the draft genome sequence of *Bacillus velezensis* CMAA1918, an osmotolerant rhizobacterium isolated from the drought-tolerant bromeliad *Neoglaziovia variegata* in the Caatinga biome. Genomic features associated with osmotic and drought-related stress responses, phosphate metabolism, siderophore production, and antimicrobial biosynthetic pathways underscore its potential relevance in water-limited agricultural systems.

## ANNOUNCEMENT

*Bacillus velezensis* strain CMAA1918 was isolated from the rhizosphere of *Neoglaziovia variegata*, a drought-tolerant bromeliad native to the Caatinga biome in northeastern Brazil. The Caatinga is one of the largest seasonally dry tropical forests (SDTFs) worldwide and is characterized by recurrent drought cycles, elevated evapotranspiration, and nutrient-poor soils (1). These abiotic constraints impose strong selective pressures that shape the root-associated microbial communities.

*B. velezensis* strains have been developed as commercial agricultural inoculants, including products, such as RhizoVital, derived from *B. velezensis* relatives and used for suppressing soil-borne diseases and enhancing plant performance (2). Additional studies have highlighted plant growth-promoting traits, rhizosphere competence, and beneficial plant–microbe interactions across strains within the *B. amyloliquefaciens–velezensis* complex (3–5). Moreover, members of this species have been investigated as probiotic organisms in aquaculture systems (6, 7). This relevance supports the characterization of new isolates from naturally stressful environments. Rhizosphere samples were collected from *Neoglaziovia variegata* in the Caatinga biome, northeastern Brazil (7°30′44.3″S, 40°07′24.2″W). Strain CMAA1918 was isolated by serial dilution and plating on nutrient agar, followed by incubation at 30°C for 48 h under aerobic conditions and purified by repeated streaking prior to genomic DNA extraction.

Genomic DNA was extracted using the Gentra Puregene kit (Qiagen), and sequencing libraries were prepared with the Illumina DNA Prep Kit. Sequencing on the NextSeq 2000 platform (2 × 100 bp) generated 7,673,029 paired-end reads (~1.5 Gb). Read quality was assessed using FastQC v0.11.9, trimming was performed with Trimmomatic v0.39, and genome assembly was conducted using SPAdes v3.13.0 on KBase, using default parameters. Genome annotation was performed with RASTtk v2.0. MiGA estimated 99.1% completeness and 4.7% contamination, and ANI analysis indicated >99% identity to *B. velezensis* reference genomes GCF_001267695.1 and GCA_001461825.1.

The draft genome assembly consists of 32 contigs, with a total genome size of 3.9 Mb and a GC content of 46.5%. The contig N50 value is 314.7 kb, with an L50 of 5, and the estimated sequencing coverage is approximately 390×. The genome encodes 7,683 predicted protein-coding genes. Genes associated with osmoprotectant uptake and metabolism, including OpuA/OpuB/OpuC systems, glycine betaine dehydrogenase, sodium–proton antiporters, and oxidative stress response were identified. Genes involved in phosphate metabolism (PhoA, PhoD), siderophore biosynthesis and uptake, and biofilm-associated regulatory systems were also detected (see Table S2 at Figshare, https://doi.org/10.6084/m9.figshare.30739667). These features align with traits commonly found in *B. velezensis* strains inhabiting root-associated habitats and environments exposed to osmotic stress. antiSMASH v8.0.4 identified 22 biosynthetic gene clusters (BGCs), including NRPS/lipopeptide, transAT-PKS, RiPP-like, and terpene classes (see Table S1 at Figshare, https://doi.org/10.6084/m9.figshare.30739667). Gene clusters corresponding to bacillaene, macrolactin H, bacillibactin, bacilysin, and a fengycin-like region (~80% similarity) were detected, consistent with biosynthetic profiles previously reported for *B. velezensis* strains (8–11). Given the documented use of *B. velezensis* strains in agriculture, the genome of CMAA1918, isolated from a drought-adapted plant microbiome, provides a resource for developing microbial inoculants. Genomic data from drought-adapted microbiomes support the identification of strains with traits that promote plant performance under hydric stress.

## Data Availability

This Whole Genome Shotgun project has been deposited at DDBJ/ENA/GenBank under the accession JBISVH000000000. The version described in this paper is version JBISVH010000000. Raw reads have been deposited in the NCBI Sequence Read Archive (SRA) under the accession SRS23112758. Tables S1 and S2 are publicly accessible for download at Figshare (https://doi.org/10.6084/m9.figshare.30739667).
